# PNPase and RhlB Interact and Reduce the Cellular Availability of Oxidized RNA in *Deinococcus radiodurans*

**DOI:** 10.1128/spectrum.02140-22

**Published:** 2022-07-20

**Authors:** Runhua Han, Jessie Jiang, Jaden Fang, Lydia M. Contreras

**Affiliations:** a McKetta Department of Chemical Engineering, The University of Texas at Austin, Austin, Texas, USA; b Institute for Cellular and Molecular Biology, The University of Texas at Austin, Austin, Texas, USA; University of Minnesota

**Keywords:** 8-oxo-7, 8-dihydroguanine, *Deinococcus radiodurans*, PNPase, RNA binding proteins, RNA oxidation, RhlB, oxidative stress

## Abstract

8-Oxo-7,8-dihydroguanine (8-oxoG) is a major RNA modification caused by oxidative stresses and has been implicated in carcinogenesis, neurodegeneration, and aging. Several RNA-binding proteins have been shown to have a binding preference for 8-oxoG-modified RNA in eukaryotes and protect cells from oxidative stress. To date, polynucleotide phosphorylase (PNPase) is one of the most well-characterized proteins in bacteria that recognize 8-oxoG-modified RNA, but how PNPase cooperates with other proteins to process oxidized RNA is still unclear. Here, we use RNA affinity chromatography and mass spectrometry to search for proteins that preferably bind 8-oxoG-modified RNA in Deinococcus radiodurans, an extremophilic bacterium with extraordinary resistance to oxidative stresses. We identified four proteins that preferably bind to oxidized RNA: PNPase (DR_2063), DEAD box RNA helicase (DR_0335/RhlB), ribosomal protein S1 (DR_1983/RpsA), and transcriptional termination factor (DR_1338/Rho). Among these proteins, PNPase and RhlB exhibit high-affinity binding to 8-oxoG-modified RNA in a dose-independent manner. Deletions of PNPase and RhlB caused increased sensitivity of D. radiodurans to oxidative stress. We further showed that PNPase and RhlB specifically reduce the cellular availability of 8-oxoG-modified RNA but have no effect on oxidized DNA. Importantly, PNPase directly interacts with RhlB in D. radiodurans; however, no additional phenotypic effect was observed for the double deletion of *pnp* and *rhlB* compared to the single deletions. Overall, our findings suggest the roles of PNPase and RhlB in targeting 8-oxoG-modified RNAs and thereby constitute an important component of D. radiodurans resistance to oxidative stress.

**IMPORTANCE** Oxidative RNA damage can be caused by oxidative stress, such as hydrogen peroxide, ionizing radiation, and antibiotic treatment. 8-oxo-7,8-dihydroguanine (8-oxoG), a major type of oxidized RNA, is highly mutagenic and participates in a variety of disease occurrences and development. Although several proteins have been identified to recognize 8-oxoG-modified RNA, the knowledge of how RNA oxidative damage is controlled largely remains unclear, especially in nonmodel organisms. In this study, we identified four RNA binding proteins that show higher binding affinity to 8-oxoG-modified RNA compared to unmodified RNA in the extremophilic bacterium *Deinococcus radiodurans*, which can endure high levels of oxidative stress. Two of the proteins, polynucleotide phosphorylase (PNPase) and DEAD-box RNA helicase (RhlB), interact with each other and reduce the cellular availability of 8-oxoG-modified RNA under oxidative stress. As such, this work contributes to our understanding of how RNA oxidation is influenced by RNA binding proteins in bacteria.

## INTRODUCTION

Reactive oxygen species (ROS) are generated either through basal cellular metabolism or from external environmental exposures to oxidative stresses, such as ionizing radiation, UV radiation, air pollution, and certain chemicals (1, 2). Importantly, increased levels of ROS can attack and damage various important cellular components, especially nucleic acids (3, 4). Both DNA and RNA oxidation are closely related to a variety of pathophysiological events, including carcinogenesis, aging, and chronic neurodegenerative diseases (5–9). In particular, compared to DNA, RNAs are more susceptible to oxidative insults given the relatively higher cellular abundance of RNA and their single-stranded nature that lacks protection offered by hydrogen bonding and specific proteins (10). Thus far, mechanisms affecting the cellular accumulation of RNA oxidation have been less characterized relative to DNA oxidation.

Among the known oxidative lesions in RNA, 7,8-dihydro-8-oxoguanosine (8-oxoG) is one of the most prevalent nucleobase oxidations (9, 11). 8-oxoG modifications on RNA can extensively interfere with translation, as well as with the base pairing of RNAs, including that of noncoding RNAs (12–14). To avoid the detrimental effects of 8-oxoG, organisms are equipped with mechanisms that eliminate oxidized RNA (15–17). As an example, the MutT protein in Escherichia coli and its mammalian homologs (such as MutT homolog 1 [MTH1]) cleave ribonucleotides carrying 8-oxoG by hydrolyzing the oxidized nucleoside diphosphates and/or triphosphates to monophosphates (18, 19), preventing the incorporation of the free oxidized nucleotides into RNA synthesis. Although the oxidized forms of RNA precursor nucleotides can be efficiently removed in such manner, 8-oxoG can also be formed in the RNA molecules already present within the cell by direct oxidation of the base (20). Unlike DNA oxidation, no dedicated repair mechanism for RNA oxidation has been identified thus far; it has been considered that oxidatively damaged RNAs are only degraded rather than repaired (21). Importantly, several RNA-binding proteins (RBPs) have been reported to preferentially bind RNAs containing 8-oxoG and contribute to the fidelity of translation in cells by eliminating the oxidized RNA away from the translational machinery (8, 15, 17, 22, 23). In mammalian cells, for example, the Auf1 protein (AU-rich element RNA-binding protein 1) preferably binds to RNA carrying one 8-oxoG residue to eliminate oxidatively damaged mRNA (24), whereas poly(C)-binding proteins PCBP1 and PCBP2 bind to more severely oxidized RNAs containing at least two 8-oxoG residues and induce the activation of apoptotic reactions (25, 26).

Relative to mammalian cells, understanding the interaction between RBPs and 8-oxoG in bacteria still lags behind. Polynucleotide phosphorylase (PNPase), a conserved 3′-5′ exoribonuclease, is the only characterized RBP that interacts with 8-oxoG-modified RNA in bacteria (27). Both E. coli PNPase and human PNPase have been shown to bind 8-oxoG-modified RNA with higher affinity than to unmodified RNA (28, 29). The preferential binding of PNPase to RNAs containing 8-oxoG suggests a role in RNA quality control; indeed, PNPase has been shown to reduce the accumulation of RNAs containing 8-oxoG and to protect cells under oxidative stress (27, 30, 31). Interestingly, PNPase can form multiprotein complexes in many bacteria (32–34). In E. coli, PNPase functions as part of the RNA degradosome complex that also contains the endoribonuclease RNase E, a DEAD-box RNA helicase RhlB, and the glycolytic enzyme enolase Eno (33). The interaction of RNase E, PNPase, and RhlB can ensure their functional cooperation and is required for an efficient RNA degradation (33). For instance, RhlB is known to unwind RNA structures in E. coli and therefore facilitates degradation by PNPase (35, 36). PNPase and RhlB can also form a complex independent of the degradosome and therefore can be involved in alternative protein-protein interactions (37). However, whether RhlB cooperates with PNPase to affect oxidized RNA under oxidative stress is still unknown.

Deinococcus radiodurans is a unique bacterium that possesses remarkable tolerance to extreme environmental conditions that generate oxidative damage to macromolecules (38). The survival and recovery capabilities of D. radiodurans postexposure to those extreme environmental stresses is remarkable, particularly considering that it undergoes high levels of RNA oxidation under some of these oxidative stresses. As an example, a significant increase of 8-oxoG-modified RNA has been observed under 15 kGy of ionizing radiation (39). However, little is known as to how oxidated RNA is managed in this organism. The 8-oxoG-recognizing protein, PNPase, is present in D. radiodurans and contributes to the survival under hydrogen peroxide (H_2_O_2_) and UV radiation (40). Interestingly, D. radiodurans also encodes many other DEAD box RNA helicases (e.g., RhlB, DR_B0136/HepA, and DR_1624) (41). However, whether and how they modulate 8-oxoG-modified RNAs in D. radiodurans is still unclear.

Here, through a combination of RNA affinity chromatography, mass spectrometry and biochemical analysis, we demonstrate that PNPase and RhlB in D. radiodurans exhibit high-affinity binding to 8-oxoG modified RNA relative to unmodified RNA in a dose-independent manner. The deletion of PNPase and RhlB significantly impairs the survival of D. radiodurans under H_2_O_2_ stress and results in elevated levels of 8-oxoG-modified RNA (not 8-oxoG-modified DNA). Most importantly, PNPase interacts with RhlB, and no additive effect on survival and 8-oxoG-RNA level was observed in the double-deletion strain of PNPase and RhlB relative to the single-deletion strains. The latter result suggests that PNPase and RhlB may function cooperatively to defend against oxidative stress in D. radiodurans. To the best of our knowledge, this is the first study that indicates the essential role of RBPs in response to cellular damage induced by oxidative stresses in D. radiodurans.

## RESULTS

### Potential 8-oxoG-binding RBPs in *D. radiodurans* are identified via RNA affinity chromatography.

**FIG 1:**
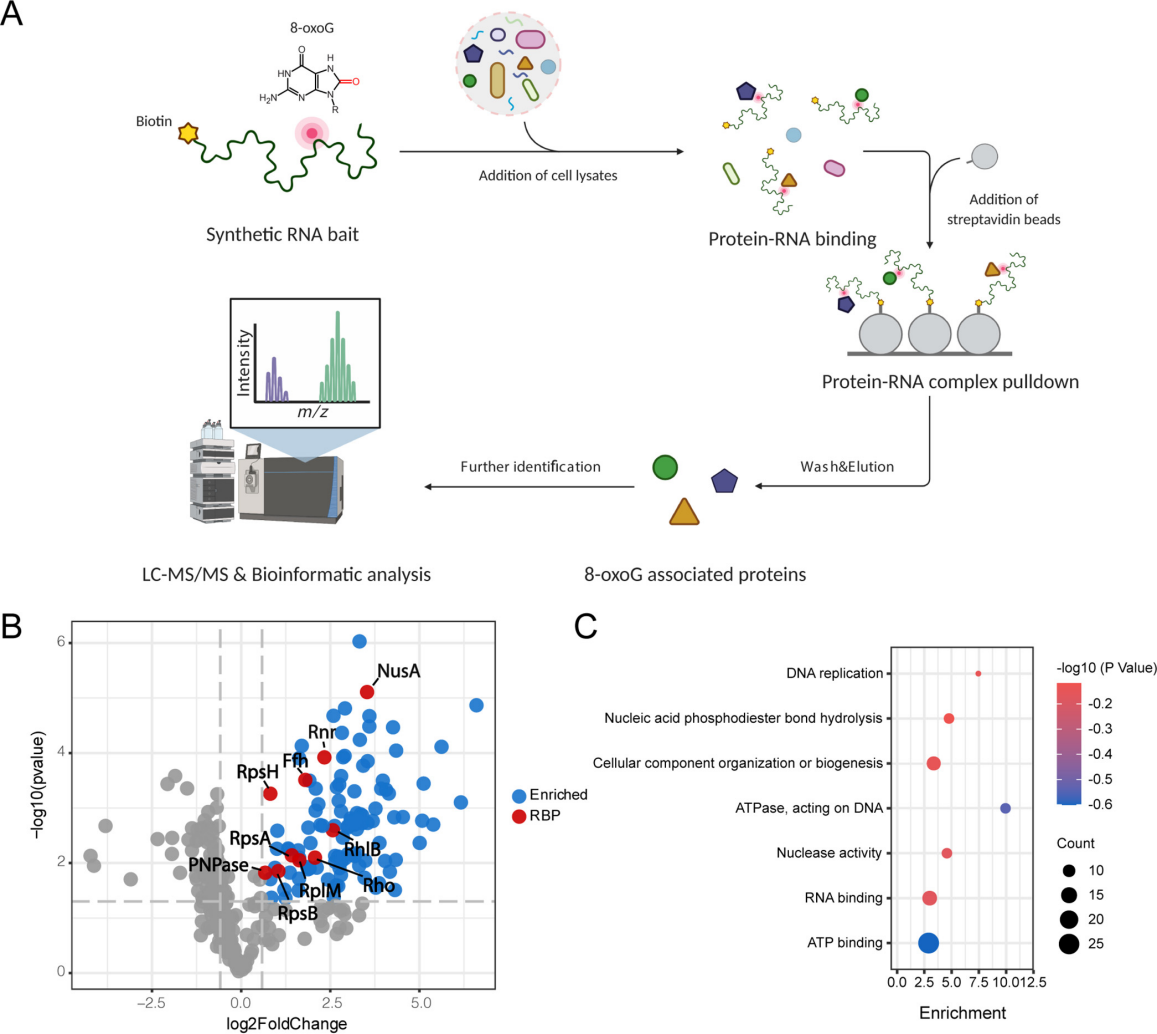
RNA affinity chromatography identifies putative 8-oxoG binding proteins in D. radiodurans. (A) The RNA affinity chromatography approach using biotinylated RNA oligonucleotides containing one 8-oxoG modification followed by LC-MS/MS and downstream analysis was used to identify novel 8-oxoG binding proteins in D. radiodurans. (B) A volcano plot shows the enrichment of proteins bound to the 8-oxoG-modified oligonucleotide versus the unmodified control oligonucleotide. Proteins that are significantly enriched (log_2_ fold change ≥ 0.58496, *P ≤ *0.05) are highlighted by blue (for those without known RNA-binding domains) or red (for those containing known RNA-binding domains) (listed in Table S1). Proteins that are not significantly enriched are represented by gray dots. (C) Gene ontology and enrichment analysis of the proteins significantly enriched in RNA affinity chromatography.

To identify RBPs that preferably bind to 8-oxoG-modified RNA in D. radiodurans, we conducted an RNA affinity chromatography approach followed by liquid chromatography-tandem mass spectrometry (LC-MS/MS) analysis (Fig. 1A). Two types of 24-mer synthetic RNA oligonucleotides were used: a bait oligonucleotide carrying an 8-oxoG modification and a control oligonucleotide carrying unmodified guanines. After incubating with cell lysates extracted from the D. radiodurans R1 strain, different proteins were pulled down using the 8-oxoG-modified and control RNA oligonucleotides, as revealed by SDS-PAGE electrophoresis and principal-component analysis (see Fig. S1A and B in the supplemental material).

Both sets of proteins eluted from the pulldown experiments were then subjected to LC-MS/MS. A total of 111 proteins were significantly enriched (log_2_ fold change ≥ 0.58496, *P ≤ *0.05) in the pulldown from the 8-oxoG-modified oligonucleotide relative to the control oligonucleotide (Fig. 1B; see also Table S1). Gene ontology and enrichment analysis showed that the proteins enriched in the pulldown using the 8-oxoG-modified oligonucleotide are involved in a variety of pathways, including DNA replication, nucleic acid phosphodiester bond hydrolysis, cellular component organization or biogenesis, and nucleotide excision repair (Fig. 1C). We also revealed a complex protein-protein interacting network among these proteins through the STRING-DB database (42) (see Fig. S1C). We suspect that the tightly connected protein-protein interaction network partially explains the high number of proteins that were identified from these assays. Notably, many proteins involved in RNA degradation and in RNA binding and nuclease activity were enriched in the 8-oxoG pulldown (see Table S1), suggesting the possibility that the interactions of these proteins with 8-oxoG-modified RNA could lead to its degradation. We further identified 10 RBPs that contain known RNA-binding domains (Fig. 1B; see also Table S1), including DR_2063/PNPase (the PNPase homolog in D. radiodurans), DR_0335/RhlB (DEAD box RNA helicase), DR_1983/RpsA (ribosomal protein S1), DR_1338/Rho (Rho transcription termination factor), DR_0353/Rnr (RNase R), DR_1797/NusA (transcription elongation factor NusA), DR_1836/Ffh (signal recognition particle protein), and other three ribosomal proteins (RplM, RpsH, and RpsB). These results suggest that these proteins could possibly directly bind to 8-oxoG-modified RNA. Interestingly, DR_0550/MutT was also found to be enriched in the 8-oxoG pulldown (see Table S1). Considering that DR_0550/MutT does not contain any known RNA-binding domain and can only recognize the oxidized RNA precursor nucleotides, not the RNA molecules oxidized after transcription (19), we believe that this might be from indirect protein-protein interaction or unspecific binding. As such, we excluded this protein from further analysis.

### PNPase, RhlB, Rho, and RpsA preferably bind to 8-oxoG oxidized RNA in *D. radiodurans*.

**FIG 2:**
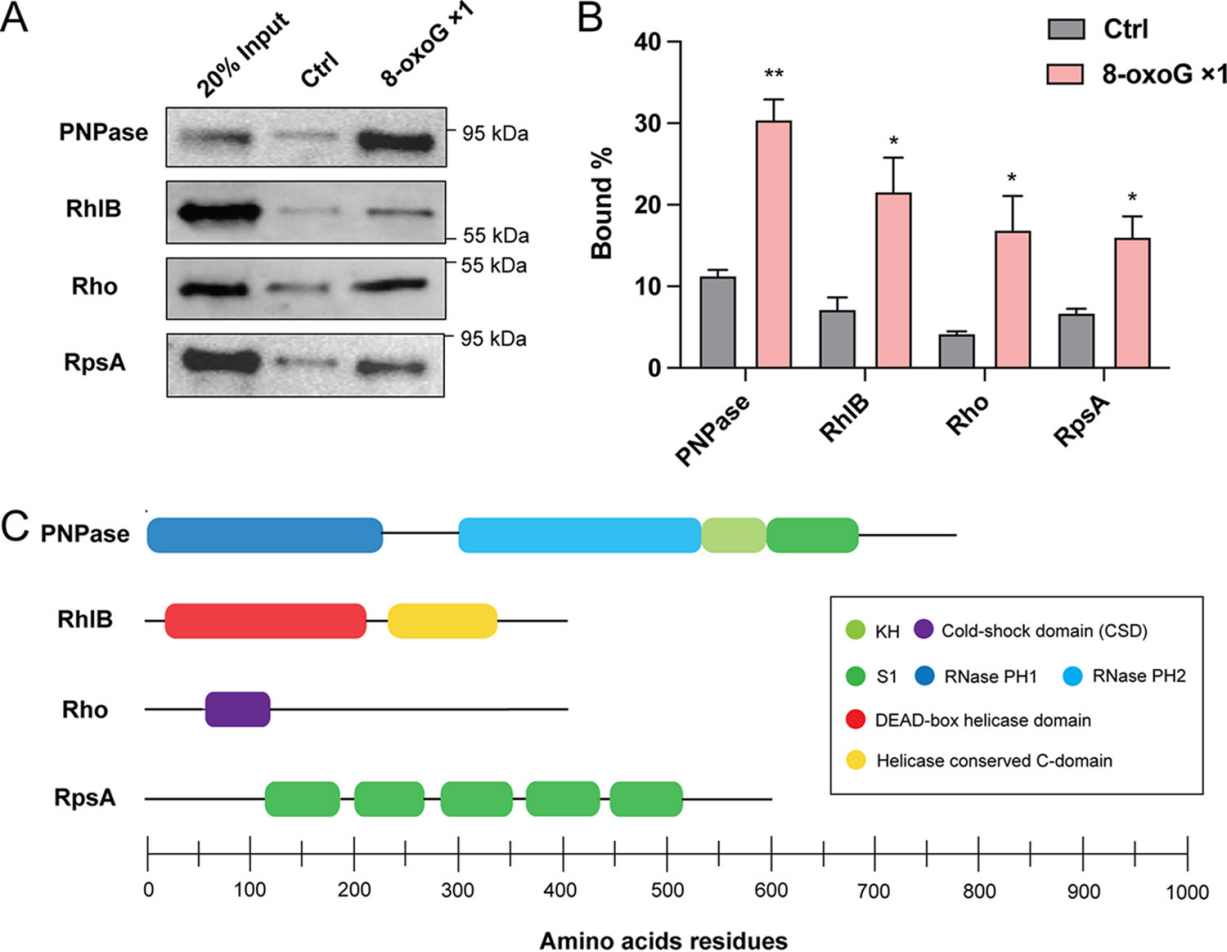
RBPs preferably bind 8-oxoG-modified oligoribonucleotide in D. radiodurans. (A) Binding of PNPase, RhlB, Rho, and RpsA to oligoribonucleotides carrying one 8-oxoG (8-oxoG ×1) and control oligoribonucleotide (Ctrl), as determined by Western blotting. 500 ng of each purified recombinant protein was incubated with 10 μg of biotinylated oligoribonucleotide carrying one 8-oxoG or no 8-oxoG and then subjected to 500 μg of Dynabead MyOne Streptavidin C1 beads. After incubation, the beads were washed, and the proteins were resuspended in SDS sample buffer. Western blotting was performed to detect the proteins in the input and the pulldown fractions with an anti-His_6_ antibody. In addition, 20% input: 20% of each recombinant protein applied to binding was also subjected to Western blotting. (B) The band intensities were measured by CLIQS (TotalLab), and the percentage bound was determined. Error bars represent the standard deviations of biological triplicates. Significance was calculated by two-tailed Student *t* test compared to Ctrl under each condition (*, *P ≤ *0.05; **, *P ≤ *0.01). (C) Domain composition of PNPase, RhlB, Rho, and RpsA. RNA-binding domains are represented as rounded boxes and color coded as indicated in the box.

To further confirm that the enrichment of these RBPs in the 8-oxoG pulldown assay reflected their preference for binding 8-oxoG-modified RNA, we purified proteins of PNPase, RhlB, RpsA, Rho, NusA, Rnr, and Ffh (RplM, RpsH, and RpsB were excluded from the further analysis given their roles in rRNA binding). Each purified protein was incubated with oligoribonucleotides carrying one 8-oxoG or no 8-oxoG; these mixtures were subjected to RNA affinity chromatography, and pulldown products were subjected to Western blotting. As shown in Fig. 2A and B, PNPase, RhlB, Rho, and RpsA exhibited selective binding to the oligoribonucleotide containing 8-oxoG compared to the control oligoribonucleotide. In contrast, Rnr and NusA showed similar binding to both oligoribonucleotides, and no Ffh protein was detected in the pulldown fractions from either oligoribonucleotide (see Fig. S2). These results indicated that, among these proteins, only PNPase, RhlB, Rho, and RpsA selectively bound 8-oxoG-modified RNA, whereas the enrichment of Ffh, Rnr, and NusA in the RNA affinity chromatography is likely from indirect protein-protein interactions (i.e., from interactions with PNPase, RhlB, Rho, or RpsA). It is worth noting that all of these proteins contain known RNA-binding domains that play diverse roles in RNA metabolisms, such as KH and S1 (43–45) (Fig. 2C), suggesting that these domains might be important for 8-oxoG recognition in D. radiodurans.

**FIG 3:**
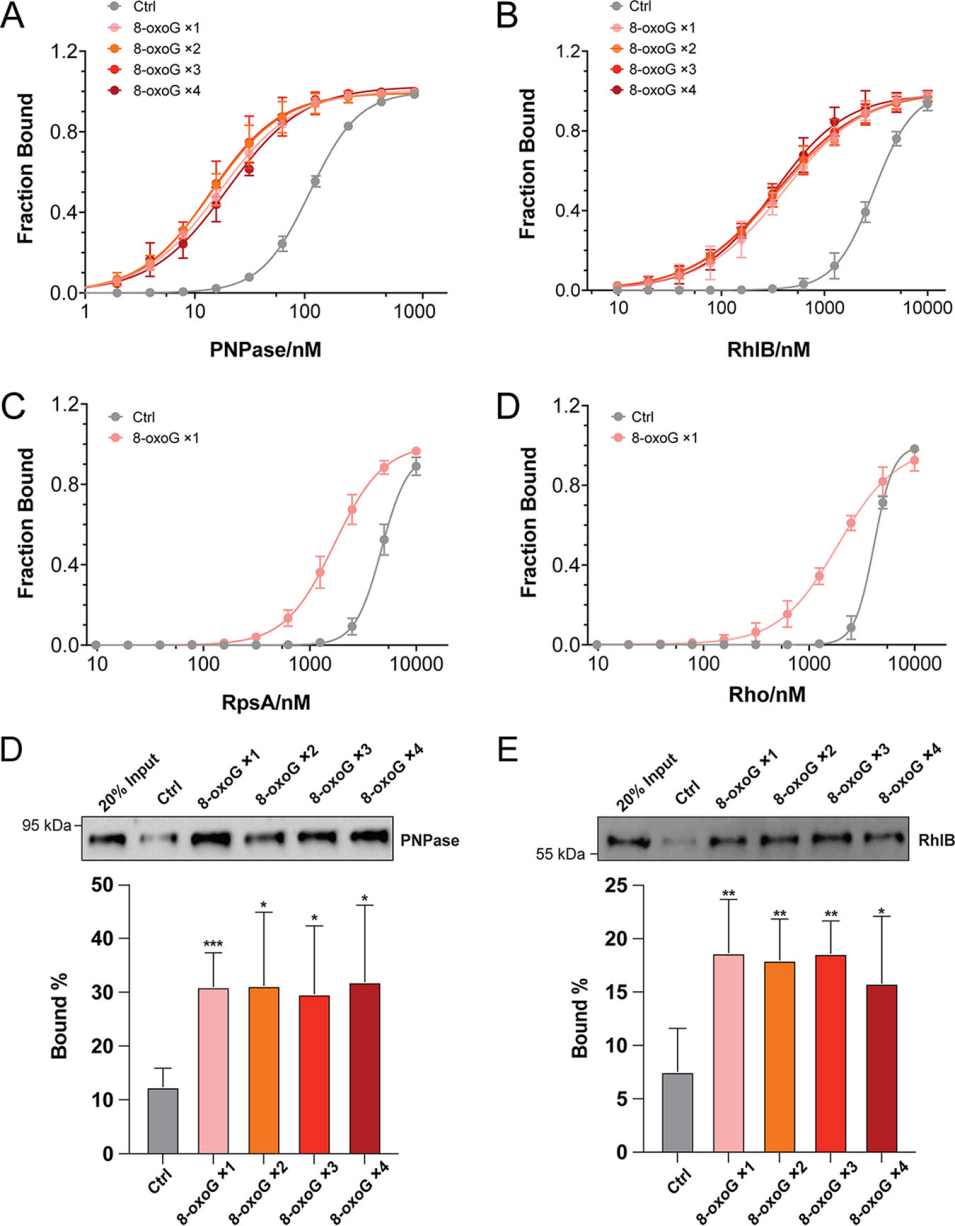
Determination of binding capability of 8-oxoG recognizing RBPs to 8-oxoG-modified and unmodified oligonucleotides. Binding affinities of PNPase (A), RhlB (B), Rho (C), and RpsA (D) to oligonucleotides carrying different numbers of 8-oxoG bases (8-oxoG ×1-4) and control oligonucleotide (Ctrl) were determined by EMSA. The dissociation constant (*K_d_*) values were measured in duplicates and are summarized in Table 1. The binding of PNPase and RhlB to oligoribonucleotides carrying 8-oxoG bases and control oligoribonucleotide was also evaluated by RNA affinity chromatography, followed by Western blotting (for PNPase [E] and RhlB [F], respectively). The band intensities shown in the bottom panels were measured by CLIQS (TotalLab), and the percentage bound was determined. Data from five independent experiments are shown. Significance was calculated by using a two-tailed Student *t* test compared to Ctrl under each condition (*, *P ≤ *0.05; **, *P ≤ *0.01; ***, *P ≤ *0.001).

We next attempted to quantify the binding affinity of the PNPase, RhlB, Rho, and RpsA to 8-oxoG-modified RNAs using electrophoretic mobility shift assays (EMSAs). In these assays, the same RNA oligoribonucleotides used in the affinity chromatography assay were radiolabeled and incubated with purified PNPase, RhlB, RpsA, and Rho at different concentrations, followed by electrophoresis in native PAGE gels. As shown in Fig. S3 in the supplemental material, all four proteins exhibited higher binding affinity to the 8-oxoG-containing oligoribonucleotide than the control oligoribonucleotide. Importantly, quantification of the dissociation constant (*K_d_*) showed that PNPase and RhlB bind to 8-oxoG-modified oligoribonucleotide with high affinity at the nanomolar range (30.0 ± 1.4 nM and 382.9 ± 47.4 nM, respectively) (Fig. 3A and B; Table 1). In contrast, RpsA and Rho showed relatively lower affinity to the 8-oxoG-modified oligoribonucleotides (1,705.1 ± 305.7 nM and 1,882.3 ± 21.2 nM) (Fig. 3C and D; Table 1). These results suggest that, among the four proteins, PNPase and RhlB are prominent RBPs that recognize 8-oxoG-modified RNA in D. radiodurans. We therefore selected these proteins for further study.

**TABLE 1:** Binding affinities of 8-oxoG-recognizing RBPs for 8-oxoG-modified oligonucleotides and unmodified control oligonucleotide^*a*^

Protein ID	Name	Mean *K_d_* (nM) ± the SD				
		Ctrl	8-oxoG ×1	8-oxoG ×2	8-oxoG ×3	8-oxoG ×4
DR_2063	PNPase	111.6 ± 7.2	30.0 ± 1.4	28.9 ± 4.2	29.4 ± 8.7	24.7 ± 1.4
DR_0335	RhlB	3,005.2 ± 137.0	382.9 ± 47.4	354.8 ± 56.7	351.2 ± 80.8	340.6 ± 47.1
DR_1983	RpsA	5,444.1 ± 403.6	1,705.1 ± 305.7	ND	ND	ND
DR_1338	Rho	3,942.2 ± 286.1	1,882.3 ± 21.2	ND	ND	ND

a Dissociation constants (*K_d_*) and standard deviations were determined in duplicate by electrophoretic mobility shift assays (Fig. 3; see also Fig. S3). 8-oxoG×1 to 8-oxoG×4, 8-oxoG-modified oligonucleotides; Ctrl, unmodified control oligonucleotide; ND, not determined.

### The binding of PNPase and RhlB to 8-oxoG-modified RNA is dose independent.

Previous studies indicate that certain RBPs may have the ability to differentiate mildly from heavily oxidized RNA in HeLa cells (25, 26). To explore whether PNPase and RhlB have a similar function, we repeated the EMSA analysis using synthetic oligoribonucleotides containing one to four 8-oxoG bases. As shown in Fig. 3A and B and Table 1, both PNPase and RhlB showed an equivalent binding affinity to the oligoribonucleotides carrying different numbers of 8-oxoGs, which is significantly higher than the binding to the control oligonucleotide without 8-oxoG. We further confirmed this result through RNA affinity chromatography assay followed by Western blotting: we found that the binding capacity of PNPase and RhlB to oligonucleotides carrying different numbers of 8-oxoGs is similar, which is significantly higher relative to the binding with the control oligonucleotide (Fig. 3E and F). These results indicate that the binding pocket of PNPase and RhlB can be saturated by only one 8-oxoG, which prevents the further binding of additional 8-oxoG bases.

### PNPase and RhlB are essential to the survival of *D. radiodurans* under H_2_O_2_ stress.

**FIG 4:**
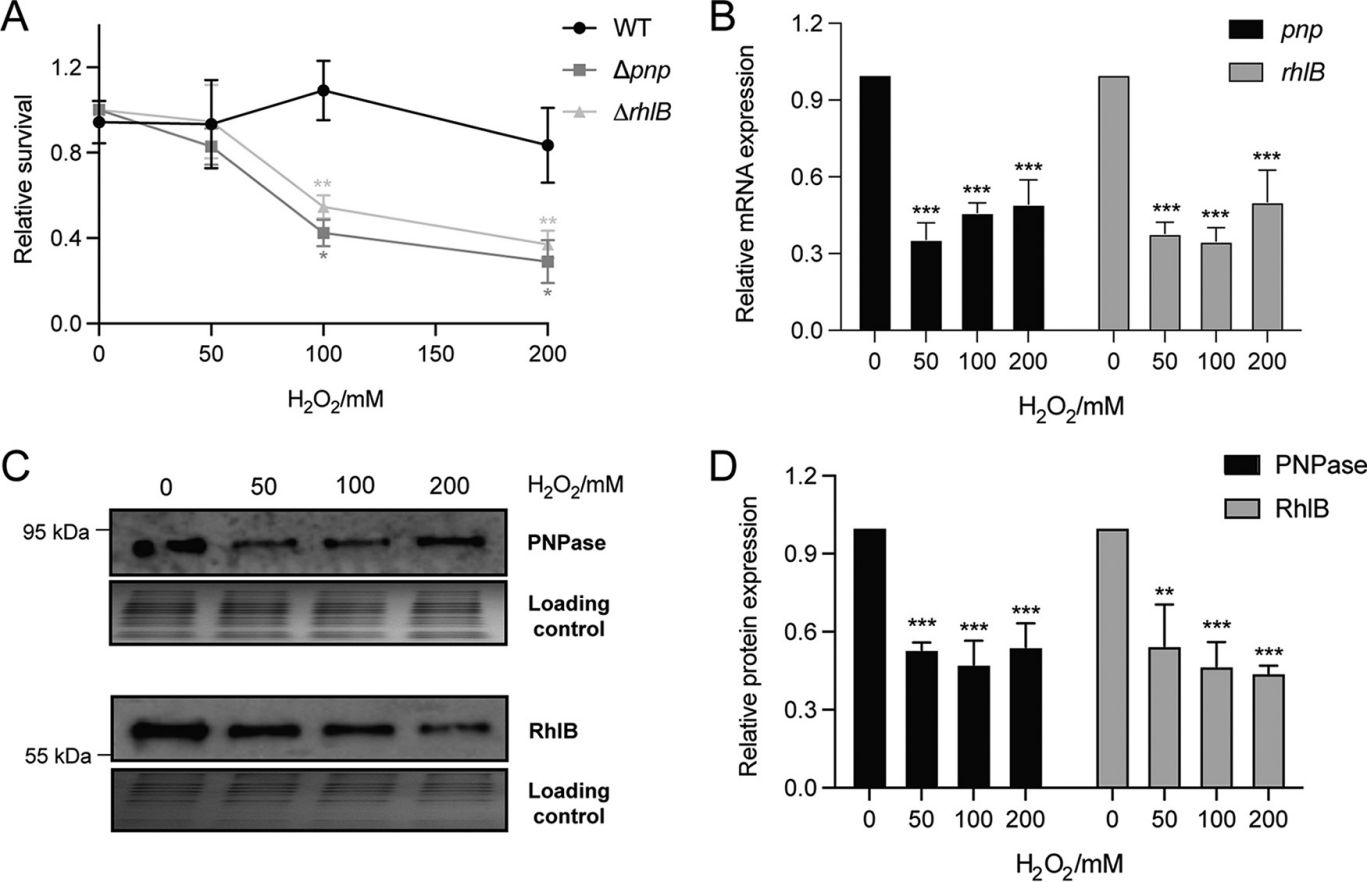
The expression and regulation of PNPase and RhlB are essential for the survival of D. radiodurans under H_2_O_2_. (A) The D. radiodurans WT, Δ*pnp*, and Δ*rhlB* strains were grown to the exponential phase and treated with 0, 50, 100, or 200 mM H_2_O_2_ for 30 min at 4°C. Bacterial survival was evaluated by plate counting after 3 days of incubation at 32°C. The data are represented as the percentages of the untreated control. (B) The mRNA levels of *pnp* and *rhlB* were determined by RT-qPCR. The D. radiodurans WT strain was grown to the exponential phase and treated with 0, 50, 100, or 200 mM H_2_O_2_ for 30 min at 4°C. The total RNA was extracted, and RT-qPCR was carried out to determine the expressions of *pnp* and *rhlB* using gene-specific primers. (C) The protein levels of PNPase and RhlB were determined by Western blotting. The D. radiodurans WT strains carrying 3×FLAG chromosomally tagged PNPase and RhlB were grown to the exponential phase and treated with 0, 50, 100, or 200 mM H_2_O_2_ for 30 min at 4°C. The total cell lysates were extracted, and Western blotting was carried out to determine the expressions of PNPase and RhlB with an anti-FLAG antibody. Protein samples were visualized by Coomassie staining in parallel to show the equivalent protein amount loaded in each lane. (D) The band intensities of Western were measured by CLIQS (TotalLab). Error bars represent the standard deviations of biological triplicates. Significance was calculated by using a two-tailed Student *t* test compared to the WT under each condition (*, *P ≤ *0.05; **, *P ≤ *0.01; ***, *P ≤ *0.001).

To further test the biological significance of PNPase and RhlB in D. radiodurans, we attempted to completely abolish the expression of these proteins in D. radiodurans using a previously described homologous recombination method (46). Full deletion strains of PNPase and RhlB were successfully constructed and confirmed by both genomic PCR (see Fig. S4A) and reverse transcription-quantitative PCR (RT-qPCR) (see Fig. S4B). The wild-type (WT), Δ*pnp*, and Δ*rhlB* strains were then exposed to 0 to 200 mM H_2_O_2_, an oxidative reagent widely used in experiments to generate oxidative RNA damage (27). The survival of these strains was assessed by serial diluting and plating. As depicted in Fig. 4A, no significant difference in survival of the WT strain was observed within the 0 to 200 mM H_2_O_2_ dosage range. In contrast, the Δ*pnp* and Δ*rhlB* strains demonstrated significantly impaired survival under 100 and 200 mM H_2_O_2_ exposure, while their survival under 0 and 50 mM H_2_O_2_ was comparable to the WT strain (Fig. 4A). Therefore, we conclude that PNPase and RhlB play crucial roles in protecting D. radiodurans cells under oxidative stress.

Importantly, significantly lower levels of PNPase and RhlB expression were detected at both mRNA and protein levels under H_2_O_2_ stress (Fig. 4B to D). It is worth noting that the absence of PNPase or RhlB does not obviously affect cell growth under the nonoxidative stress condition (see Fig. S4C); these results imply that PNPase and RhlB may be more important under oxidative stress conditions relative to non-oxidative stress conditions in D. radiodurans. In addition, deletions of PNPase and RhlB do not affect the intracellular H_2_O_2_ level (see Fig. S4D), which is contrary to the function of catalase (encoded by *katA*), the main antioxidant protein in D. radiodurans that removes H_2_O_2_ from the cells efficiently under oxidative stress (see Fig. S4D). These results suggest that the reduced cell viability of the Δ*pnp* and Δ*rhlB* strains is not due to deficient bacterial growth or reduced H_2_O_2_ in the cell culture.

### PNPase and RhlB reduce the cellular availability of 8-oxoG in RNA under H_2_O_2_ stress.

**FIG 5:**
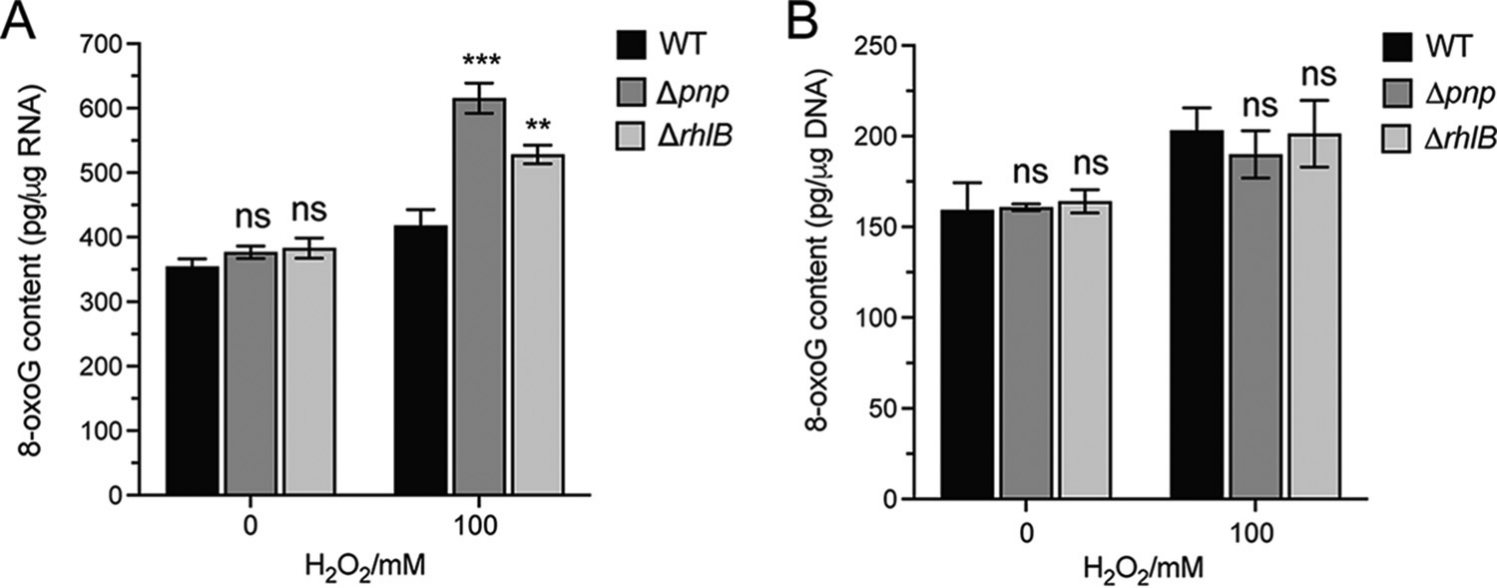
PNPase and RhlB reduce the cellular availability of 8-oxoG-RNA in D. radiodurans under H_2_O_2_. The D. radiodurans WT, Δ*pnp*, and Δ*rhlB* strains were grown to the exponential phase and treated with 0 or 100 mM H_2_O_2_ for 30 min at 4°C. Total RNA or DNA was extracted, digested, and subjected to ELISA to determine 8-oxoG-RNA (A) and 8-oxoG-DNA (B) levels.

Given that both PNPase and RhlB showed preferential binding to 8-oxoG-modified RNA, we hypothesized that they may be involved in processing 8-oxoG-modified RNAs in the cells under oxidative stress. To test this, we determined whether the concentration of 8-oxoG-modified RNA is affected *in vivo* by PNPase or RhlB deletion. The WT, Δ*pnp*, and Δ*rhlB* strains were challenged with 0 and 100 mM H_2_O_2_ for 30 min, followed by RNA extraction and enzyme-linked immunosorbent assays (ELISAs) using a specific 8-oxoG antibody. As shown in Fig. 5A, significantly higher concentrations of 8-oxoG-modified RNA were detected upon H_2_O_2_ exposure relative to no treatment in all strains. Notably, the levels of 8-oxoG-modified RNA detected in all three strains in the absence of H_2_O_2_ exposure were comparable. However, increased levels of 8-oxoG-modified RNA were detected in the Δ*pnp* and Δ*rhlB* strains under H_2_O_2_ compared to the WT strain (Fig. 5A). Importantly, we confirmed the absence of DNA contamination in the RNA samples (see Fig. S4E), suggesting the 8-oxoG changes detected in the ELISAs were not from the DNA contamination in the samples. These results suggest that the expression of PNPase and RhlB can reduce the cellular availability of 8-oxoG-modified RNA.

Given that DNA can also be severely damaged under oxidative stress (9, 10), we further examined whether PNPase and RhlB can also affect DNA oxidation. Interestingly, we found that the levels of 8-oxoG-modified DNA in the Δ*pnp* and Δ*rhlB* strains are not significantly different compared to the WT strain under both 0 and 100 mM H_2_O_2_ treatment (Fig. 5B). These results suggest that PNPase and RhlB are only required to modulate the cellular availability of 8-oxoG-modified RNA under oxidative stress but have little effect on DNA oxidation. These results also indicate that the reduced viability of the Δ*pnp* and Δ*rhlB* mutant strains under H_2_O_2_ may be related to the accumulation of oxidated RNA (more so than to the accumulation of oxidized DNA).

### PNPase interacts with RhlB and RpsA in *D. radiodurans*.

**FIG 6:**
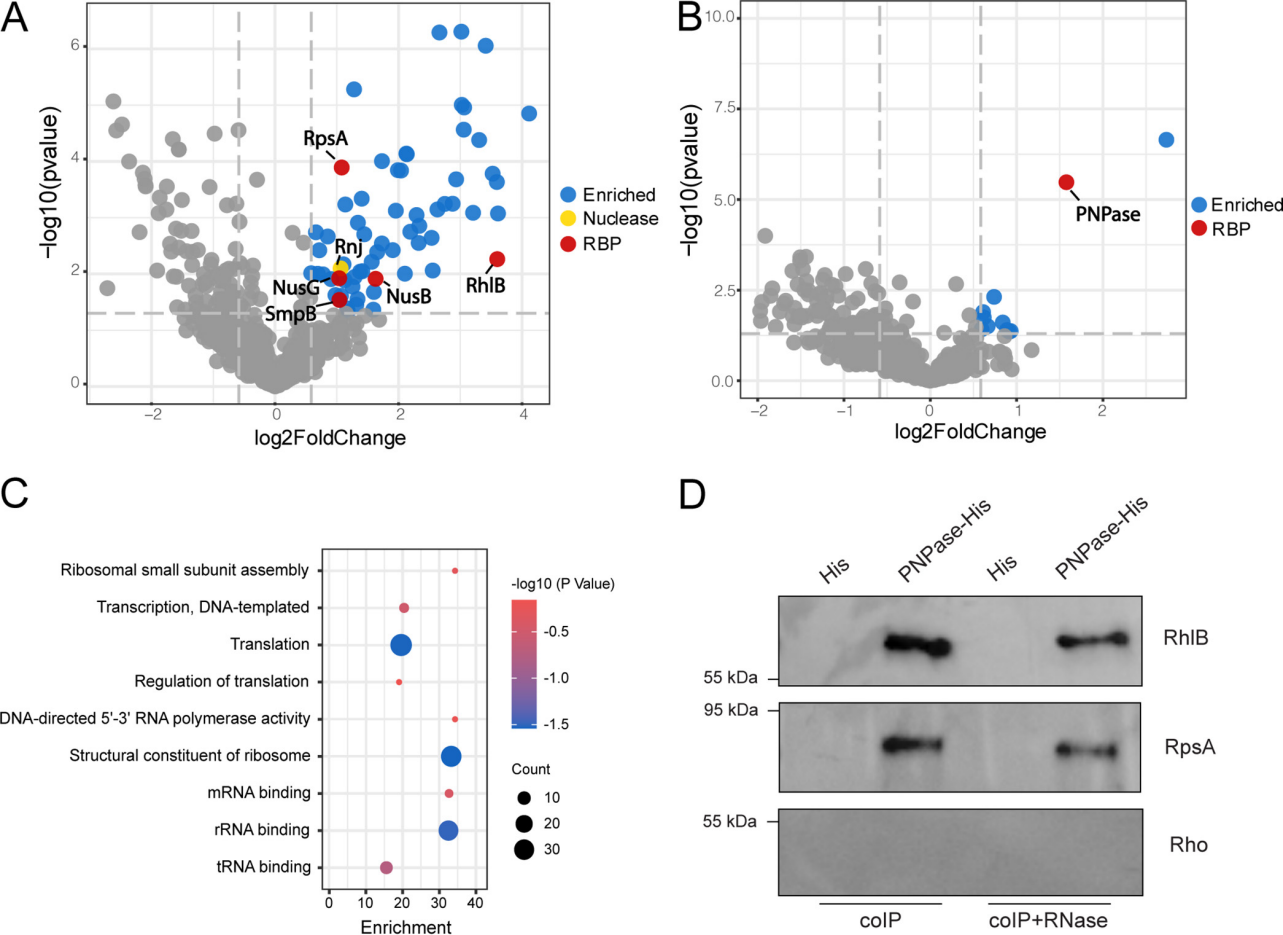
PNPase interacts with RhlB and RpsA in D. radiodurans. Volcano plots show the enrichment of proteins in the PNPase-coIP (A) and RhlB-coIP (B). Proteins that are significantly enriched (log_2_ fold change ≥0.58496, *P ≤ *0.05) are highlighted by blue (for those without known RNA-binding domains), red (for those containing known RNA-binding domains), or gold (for those with known nuclease activity) (listed in Table S1). Proteins that are not significantly enriched are represented by gray dots. (C) Gene ontology and enrichment analysis of the proteins significantly enriched in the PNPase-coIP. (D) Confirmation of the interaction of PNPase with RhlB and RpsA. His_6_ tag or His_6_-tagged PNPase on pRADgro plasmid was introduced into D. radiodurans wild-type strains carrying 3×FLAG chromosomally tagged RhlB, RpsA, or Rho. After cross-linking and sonication, 500-μg of cell lysates were treated with or without RNase A and T1 and then incubated with 50 μL of Ni-NTA magnetic beads. After a washing step, Western blotting was performed to detect the proteins in the pulldown fractions using an anti-FLAG antibody.

It is worth noting that PNPase can interact with RhlB and RpsA in E. coli, either forming an individual complex or as part of the degradosome (36, 47). However, the contribution of these interactions in D. radiodurans to oxidative RNA damage, particularly as part of the oxidative stress response remains to be elucidated. To investigate whether PNPase could interact with other 8-oxoG binding proteins in D. radiodurans, we expressed C-terminus His_6_-tagged PNPase or RhlB on the pRADgro plasmids as baits and used the WT D. radiodurans strain expressing only the His_6_ tag on the pRADgro plasmid as the control. We then performed protein-protein coimmunoprecipitation (coIP) experiments, followed by LC-MS/MS analysis, and 584 and 552 proteins were identified in PNPase- and RhlB-coIP experiments, respectively. Among these proteins, 66 proteins were significantly enriched (log_2_ fold change ≥ 0.58496, *P ≤ *0.05) in the PNPase-coIP compared to the control strain (Fig. 6A; see also Table S2). In contrast, only 11 proteins were significantly enriched (log_2_ fold change ≥ 0.58496, *P ≤ *0.05) in the RhlB-coIP (Fig. 6B; see also Table S2), suggesting the possibility that this protein is involved in a smaller number of protein-protein interactions in D. radiodurans. The proteins enriched in the PNPase-coIP include DNA repair proteins (e.g., RecA and Ssb), DNA-directed RNA polymerase subunits (RpoA, RpoB, and RpoC), as well as many ribosomal proteins (see Table S2). Gene ontology and enrichment analysis revealed that these proteins are involved in diverse pathways, especially ribosome assembly/biogenesis, transcription, and translation processes (Fig. 6C). These proteins are also involved in binding to tRNA, mRNA, and rRNA (Fig. 6C). However, it is also possible that (given their abundance) some ribosomal proteins were purified with PNPase due to nonspecific interactions, which has been observed previously (48). Overall, our results from the PNPase-coIP indicate that PNPase participates in large protein-protein networks that likely play important roles in D. radiodurans. This is further supported by the STRING-DB analysis showing that 87.88% of these proteins (58 of 66) are potentially interacting with each other (see Fig. S5). Importantly, interactions between 8-oxoG recognizing RBPs were also observed: we identified RhlB and RpsA in the coIP of PNPase (Fig. 6A; see also Table S2) and PNPase in the RhlB-coIP (Fig. 6B; see also Table S2). These data are consistent with the previous studies in E. coli (33, 44), and support a model where RhlB or RpsA may form a complex with PNPase to process 8-oxoG-modified RNA in D. radiodurans.

We further confirmed the direct interaction of PNPase with RhlB and RpsA in D. radiodurans through Western blotting. Protein-protein coIP experiments were performed using the C-terminal His_6_-tag PNPase on the pRADgro plasmid as a bait. The anticipated/potential interaction partner (RhlB, RpsA, or Rho) was tagged with 3×FLAG at the C terminus on the chromosome to allow detection by Western blotting with an anti-FLAG antibody. As shown in Fig. 6D, RhlB-3×FLAG and RpsA-3×FLAG were successfully copurified with the strain carrying plasmid expressed His_6_-tagged PNPase. Notably, these copurifications were not abolished when cell lysates were treated with RNases, suggesting that the interaction between PNPase with RhlB or RpsA does not rely on their RNA substrates (Fig. 6D). On the contrary, no Rho-3×FLAG was detected in the PNPase-coIP from either RNase-treated or untreated lysates (Fig. 6D). Altogether, our results revealed active interaction between PNPase with RhlB and RpsA in D. radiodurans in an RNA-independent manner.

### RhlB and PNPase double deletion in *D. radiodurans* shows an effect similar to single deletions.

**FIG 7:**
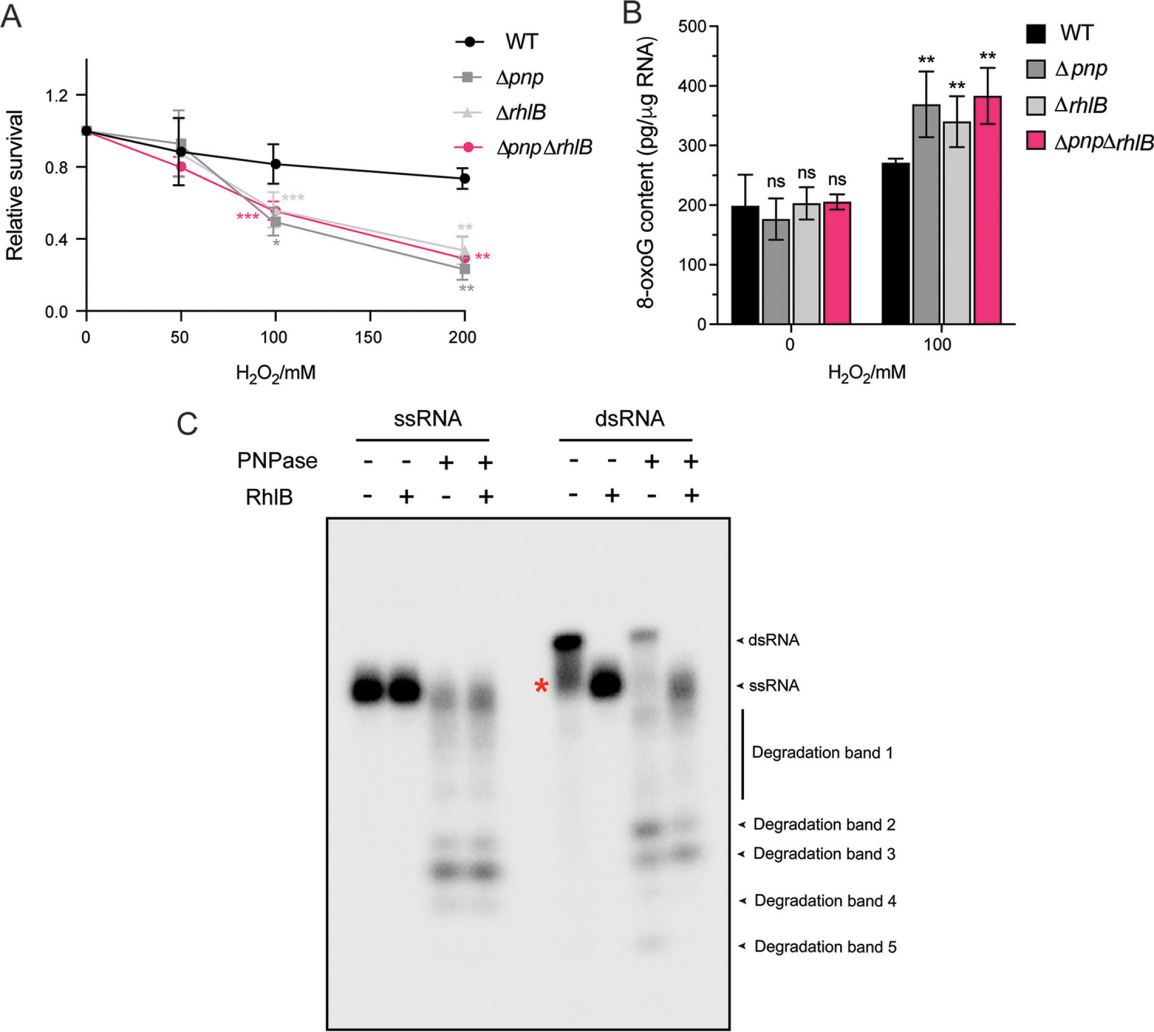
The double-deletion strain of PNPase and RhlB exhibits an effect similar to that for single-deletion strains in D. radiodurans. (A) D. radiodurans WT, Δ*pnp*, Δ*rhlB*, and Δ*pnp *Δ*rhlB* strains were grown to the exponential phase and treated with 0, 50, 100, or 200 mM H_2_O_2_ for 30 min at 4°C. Bacterial survival was evaluated by plate counting after 3 days of incubation at 32°C. The data are represented as percentages of the untreated control. (B) The 8-oxoG-RNA levels in WT, Δ*pnp*, Δ*rhlB*, and Δ*pnp *Δ*rhlB* strains treated with 0 or 100 mM H_2_O_2_ were determined by ELISA. Error bars represent the standard deviations of biological triplicates. Significance was calculated by using a two-tailed Student *t* test compared to the WT under each condition (**, *P ≤ *0.01; ***, *P ≤ *0.001). (C) RhlB promotes PNPase degradation on dsRNA in D. radiodurans. A synthesized 22-mer ssRNA was radiolabeled at 5′ end and incubated with a short (11-mer) RNA oligonucleotide to form the hybridized dsRNA. RNA degradation and unwinding were evaluated by adding PNPase or RhlB alone or both proteins to ssRNA or dsRNA, followed by 30 min of incubation at 32°C. Reaction products were resolved in a 20% native polyacrylamide gel. The remaining ssRNA residue after hybridization is labeled by a red asterisk. The degradation bands were also labeled.

Given that PNPase and RhlB show the strongest binding affinity to 8-oxoG-modified RNA among the four 8-oxoG recognizing RBPs (Table 1) and that we were able to confirm their effects on oxidized RNA *in vivo* (Fig. 4 and 5). We further tested whether the association of PNPase with RhlB is important for their functional roles in D. radiodurans under oxidative stress. To achieve this, a double deletion strain of *pnp* and *rhlB* (Δ*pnp*Δ*rhlB*) was constructed, and the response of this strain to the H_2_O_2_ stress was evaluated. We found that the Δ*pnp*Δ*rhlB* strain exhibited similar survival compared to single-deletion strains (Δ*pnp* and Δ*rhlB*) under H_2_O_2_ stress, which is significantly lower than WT under 100 and 200 mM H_2_O_2_ (Fig. 7A). In addition, the accumulation of 8-oxoG-RNA in the Δ*pnp*Δ*rhlB* strain was also comparable with that in Δ*pnp* and Δ*rhlB* strains under 100 mM H_2_O_2_ (Fig. 7B). These results imply that these two proteins may have redundant activities or function together in modulating RNA oxidation to protect D. radiodurans from oxidative stress.

RhlB is an RNA helicase that unfolds structured RNA to promote the degradation of the substrate by PNPase in the E. coli (36). To identify the functional role of the PNPase-RhlB interaction in D. radiodurans, we examined the degradation of single RNA (ssRNA) and double-stranded RNA (dsRNA) by PNPase *in vitro*. We used a 22-mer ssRNA and an artificial dsRNA as the substrates in PNPase degradation assays in the presence or absence of RhlB. As depicted in Fig. 7C, PNPase can degrade ssRNA, resulting in several bands (degradation bands 1 to 4 in Fig. 7C) but had little effect on dsRNA in the absence of RhlB. However, degradation of the dsRNA reached the same level of ssRNA when RhlB was added to the reaction (Fig. 7C), indicating that the unwinding ability of RhlB can aid the degradation of dsRNA by PNPase in D. radiodurans. The partial degradation of the dsRNA when only PNPase was added (degradation bands 1 to 5 in Fig. 7C) may result from the remaining presence of ssRNA (indicated by the red asterisk in Fig. 7C, which we assume is still present in the dsRNA reaction) due to incomplete annealing of the single RNA chain since the hybridization efficiency was not 100%. These results suggest that PNPase and RhlB may function cooperatively to degrade structured RNA in D. radiodurans. It is worth noting that we also attempted to conduct this assay using oligonucleotides carrying 8-oxoG. Unfortunately, no dsRNA can be observed using 8-oxoG-modified oligonucleotides; the presence of 8-oxoG might affect the formation of dsRNA by impairing the base-pairing during RNA-RNA hybridization (14, 49). Further studies are needed to further confirm whether the RhlB can promote the degradation of structured oxidized RNAs.

## DISCUSSION

In the past, extensive studies have been conducted to understand the mechanism of the resistance of D. radiodurans to oxidative stresses, most of which mainly focused on gene regulation, protein functions, and sRNA regulations (38, 39, 46, 50–52). Our previous work has revealed that ionizing radiation can cause a significantly increased 8-oxoG-RNA concentration in D. radiodurans (39). However, the contribution of molecular systems that affect the processing of RNA modifications in the context of oxidative stress resistance of D. radiodurans is limited. Here, we further showed that D. radiodurans possess several RBPs (particularly PNPase and RhlB) that can preferably bind and affect the cellular availability of the 8-oxoG-modified RNA to protect cells under oxidative stress; this represents another mechanism underlying the extreme resistance of D. radiodurans to oxidative stresses.

8-oxoG modification in RNA can cause a decrease in the fidelity of gene expression or translation and is closely related to a range of diseases (5, 14, 20). Based on previous studies, 8-oxoG-modified RNA can be recognized by RBPs and further processed via pathways related to RNA decay or programmed cell death (8, 15, 17, 22, 23). Several proteins that exhibit selective binding to RNA molecules containing 8-oxoG have been uncovered, such as Auf1, PCBP1, PCBP2, and PNPase (24, 25, 29). However, most of these proteins have been characterized in model organisms (E. coli and HeLa cells).

In the present study, we discovered a set of RBPs (PNPase, RhlB, RpsA, and Rho) that selectively bind 8-oxoG-modified RNA in the nonmodel bacterium D. radiodurans. Given that only PNPase was previously shown to exhibit preferential binding to 8-oxoG-modified RNA (28, 29), these data indicate that RhlB, RpsA, and Rho in D. radiodurans may also have similar functions. However, unfortunately, we were not able to confirm the importance of RpsA and Rho in controlling RNA oxidation since deletion of these proteins could not be achieved, likely due to their critical roles for cell viability in D. radiodurans. Importantly, through EMSA analysis, we determined that PNPase and RhlB showed much higher binding affinities to 8-oxoG-modified RNA than RpsA and Rho (Table 1), implying that PNPase and RhlB may play prominent roles in combating RNA oxidation in D. radiodurans. Therefore, we only focused on PNPase and RhlB in this study. Interestingly, PNPase and RhlB bind to 8-oxoG-modified RNA in a dose-independent manner, which was not observed for other 8-oxoG binding proteins (e.g., Auf1 in HeLa cells showed dose-dependent binding on 8-oxoG-modified oligonucleotides) (26). It is possible that the binding pocket of PNPase and RhlB can be saturated by a single 8-oxoG, which prevents the further binding of additional 8-oxoG bases. However, the underlying mechanism requires further investigation.

The preferential binding of PNPase and RhlB to 8-oxoG-modified RNA relative to unmodified RNA suggests their important roles in 8-oxoG-RNA processing and oxidative stress response in D. radiodurans. Indeed, deletion of PNPase and RhlB resulted in reduced survivability of D. radiodurans and elevated 8-oxoG-RNA levels upon H_2_O_2_ exposure (Fig. 4A and Fig. 5A). On the contrary, there is no change of 8-oxoG-DNA when the expression of the two proteins was abolished (Fig. 5B), suggesting that the reduced viability of the Δ*pnp* and Δ*rhlB* mutant strains under H_2_O_2_ may only be related to the accumulation of oxidized RNA in the cells (not oxidized DNA). Notably, the expression of PNPase and RhlB were observed to significantly decrease at both mRNA and protein levels under H_2_O_2_ stress (Fig. 4B to D); a similar phenomenon has been observed with human 8-oxoG recognizing RBPs (53). The decreased expression of PNPase and RhlB under H_2_O_2_ stress might be beneficial to other functional roles of these proteins, such as sRNA regulations and RNA decay (54–56). It is also worth noting that the impact of PNPase and RhlB seems more significant under high level of oxidative stress (Fig. 4A and Fig. 5A; see also Fig. S4C). In contrast, their effect under normal physiological conditions is little (Fig. 4A and Fig. 5A; see also Fig. S4C), when antioxidant proteins (i.e., catalase) are sufficient to combat the adverse effects caused by the lower (basal) level of oxidative stress.

Importantly, it is known that the degradation activity of PNPase can be impaired by secondary structures (e.g., stem-loop structures) occurring internally or at the 3′ end of the RNA substrates (57). Combining proteomics and biochemical approaches, we identified that PNPase associates with RhlB in D. radiodurans independent of RNA (Fig. 6D). Further, both PNPase and RhlB are important for preventing the accumulation of 8-oxoG-modified RNA and for the survival of D. radiodurans under oxidative stress (Fig. 4 and 5), and the double-deletion experiments of the two proteins indicate that they may function cooperatively to eliminate the 8-oxoG-modified RNA (Fig. 7A and B). In addition, RhlB facilitates PNPase’s degradation on dsRNA *in vitro* (Fig. 7C). Based on these results, we speculate that RhlB may selectively capture structured RNAs that are modified with 8-oxoG and break down the secondary structures to enable PNPase’s functions in D. radiodurans. Unfortunately, we were not able to confirm this hypothesis since no dsRNA can be observed using 8-oxoG-modified oligonucleotides since 8-oxoG interferes with the base-pairing and potentially affects the dsRNA formation (14, 49). Moreover, despite the fact that PNPase has been demonstrated to preferably bind 8-oxoG-modified RNA in numerous organisms (28, 29), in this study we cannot distinguish whether PNPase/RhlB modulates the cellular availability of 8-oxoG-modified RNAs by direct and/or indirect degradation or by sequestration. We also cannot exclude the possibility that PNPase and RhlB exhibit redundant roles or act at different levels in modulating RNA oxidation. The investigation into these topics will need to be performed in future work.

Lastly, our study reveals the interactions of PNPase with many other proteins in D. radiodurans, including DNA repair proteins, and RNA polymerase subunits (Fig. 6A; see also Table S2). Although some of the proteins identified from the PNPase-coIP experiment may result from indirect protein-protein interactions, it is very likely that PNPase participates in a large protein network in D. radiodurans, cooperatively functioning with other proteins in multiple pathways. Interestingly, RpsA (the ribosomal protein S1) was also found to interact with PNPase in D. radiodurans in an RNA-independent fashion (Fig. 6D), suggesting that PNPase may also interact with RpsA to form a complex to cope with the oxidized RNA. Among many of the PNPase-involved protein complexes, the RNA degradosome is the most famous; it shows wide compositional variation across different bacteria (33). Thus far, the composition and function of the degradosome in D. radiodurans are still unknown. In our coIP data, we found that RhlB and RNase J (DR_2417/Rnj) can also be copurified with PNPase in D. radiodurans (Fig. 6A; see also Table S2), indicating that RNase J or RhlB may function as part of the D. radiodurans degradosome together with PNPase.

Altogether, our work provides new insights into how D. radiodurans scavenges oxidized RNA molecules under oxidative stress and significantly expands our knowledge of how RBPs recognize and eliminate oxidized RNA in bacteria. In our future studies, we also hope to explore the location of the 8-oxoG modifications in the transcriptome of D. radiodurans and analyze how the modification locations contribute to the detoxification of RNA oxidation.

## MATERIALS AND METHODS

### Strains and culture conditions.

The D. radiodurans strain R1 (ATCC 13939) and its derivatives were cultured aerobically in TGY media (1% tryptone, 0.1% glucose, 0.5% yeast extract) at 32°C or on agar plates when required. E. coli DH10b and BL21(DE3) strains were grown aerobically in Luria-Bertani (LB) media (10 g/L tryptone, 10 g/L NaCl, and 5 g/L yeast extract) or plates at 37°C. All strains used in this study are listed in Table S3. When necessary, antibiotics were used at the following concentrations: ampicillin at 100 μg/mL for E. coli, chloramphenicol at 15 μg/mL for E. coli and 3.4 μg/mL for D. radiodurans, and kanamycin at 50 μg/mL for E. coli and 16 μg/mL for D. radiodurans.

### Construction of plasmids and strains.

The D. radiodurans mutants were constructed by double crossover recombination of a kanamycin resistance cassette into the genome to replace the intended mutation region as described previously (46). Gene tagging with 3×FLAG was achieved by replacing the stop codon with a 3×FLAG coding sequence using the same strategy. To construct the D. radiodurans strains expressing C-terminal His_6_-tagged PNPase and RhlB, the coding sequences were amplified and cloned into the SacII- and BamHI-cut sites after *groES* promoter on the pRADgro plasmid (50). The reverse primers contain CACCACCACCACCACCAC at the 5′ end to replace the stop codon. The strain expressing His_6_ was constructed by inserting CACCACCACCACCACCAC between the same cut sites on the pRADgro plasmid using Gibson assembly. The recombinant plasmids were transformed into D. radiodurans strains, and the resulting colonies were selected on TGY plates with chloramphenicol. For the construction of plasmids expressing His-tagged proteins, the coding region of each protein was amplified and cloned into the NdeI- and BamHI-cut sites on pET28a plasmid so that the His_6_ tag can be added to the N-terminal ends of the proteins for purification. The resultant plasmids were then electrotransformed into the E. coli BL21(DE3) strains. The colonies were then confirmed by PCR screening and Sanger sequencing. All plasmids and primers used in this study are listed in Table S3 and S4, respectively.

### RNA affinity chromatography.

To identify specific 8-oxoG binding proteins, a 24-mer RNA oligonucleotide (8-oxoG ×1; 5′-biotin-NNNGNNNNNGNNNNN8-oxoGNNNNNGNN-3′; N stands for a random base [A/T/C/G]) was chemically synthesized for the RNA affinity chromatography assay, and an unmodified oligonucleotide (Ctrl; 5′-biotin-NNNGNNNNNGNNNNNGNNNNNGNN-3′) was used as a control. To determine whether the binding of PNPase and RhlB is dose dependent, three more RNA oligonucleotides containing different numbers of 8-oxoG were used: 8-oxoG ×2 (5′-biotin-NNNGNNNNNGNNNNN8-oxoGNNNNN8-oxoGNN-3′), 8-oxoG ×3 (5′-biotin-NNNGNNNNN8oxoGNNNNN8-oxoGNNNNN8-oxoGNN-3′), and 8-oxoG×4 (5′-biotin-NNN8oxoGNNNNN8oxoGNNNNN8-oxoGNNNNN8-oxoGNN-3′). Next, 100 mL of D. radiodurans cells (in biological triplicates) was harvested at an optical density at 600 nm (OD_600_) of 0.8, washed, and resuspended in lysis buffer (1 mM Tris-HCl [pH 8.0] containing 1 mM phenylmethylsulfonyl fluoride [PMSF]). The cells were then lysed using sonication as described before (46). Next, the cell extracts were collected by centrifugation at 12,000 × *g* to obtain whole-cell lysates from the supernatant, and the concentrations were measured by using the Bradford assay (Bio-Rad). Then, 500 μg of the lysates or purified His_6_-tagged proteins were precleared with 500 μg of Dynabead MyOne Streptavidin C1 (Thermo Fisher Scientific) in 300 μL of binding buffer (10 mM Tris-Cl [pH 7.5], 150 mM KCl, 1.5 mM MgCl_2_, 0.05% [vol/vol] IGEPAL CA-630) for 30 min at 4°C with rotation in the presence of 0.4 U/mL SUPERase-In RNase Inhibitor (Thermo Fisher Scientific) and 125 μg of yeast tRNA (Sigma-Aldrich). Then, 10 μg of biotinylated modified/unmodified RNA baits in 20 mM Tris-Cl (pH 7.5) was refolded by heating at 90°C for 10 min, followed by incubation with precleared lysates or His_6_-tagged protein (200 μg) for 30 min at room temperature and then for 2 h at 4°C. The mixture was then added to 500 μg of Dynabeads MyOne Streptavidin C1 preblocked with BSA (1%) and tRNA (50 μg/mL) for 2 h at 4°C. The RNA-protein complexes were pulled down and washed with 900 μL of wash buffer (10 mM Tris-Cl [pH 7.5], 100 mM NaCl, 150 mM KCl, 1.5 mM MgCl_2_, 0.05% [vol/vol] IGEPAL CA-630) three times. The bead pellets were then eluted using 20 μL of high-salt elution buffer (10 mM Tris-Cl [pH 7.5], 500 mM NaCl, 150 mM KCl, 1.5 mM MgCl_2_, 0.05% [vol/vol] IGEPAL CA-630). To increase the yield, the elution step was repeated, and the eluates were combined. The eluted proteins were then analyzed by SDS-PAGE, Western blotting, or LC-MS/MS.

### Electrophoretic mobility shift assays.

N-terminal His_6_ tagged proteins were purified from the soluble lysate using Ni-NTA agarose (Qiagen) as described previously (58). EMSAs were performed to determine the binding affinity of the proteins to 8-oxoG-modified and unmodified RNA *in vitro*, as described previously (59). The oligonucleotides used in the affinity chromatography assays (same compositions except with no biotin on the 5′ end) were radiolabeled with 10 μCi of [γ-^32^P]ATP (Perkin-Elmer) by T4 polynucleotide kinase (New England BioLabs) and purified using an Oligo Clean & Concentrator kit (Zymo Research). Next, 10 fmol of radiolabeled RNA probes (at a final concentration of 1 nM) was incubated with increasing amounts of each purified proteins (0 to 10,000 nM, final concentration) in 10 μL reactions in 1× binding buffer (20 mM Tris-HCl [pH 8.0], 1 mM MgCl_2_, 20 mM KCl, 10 mM Na2HPO4-NaH_2_PO_4_ [pH 8.0], and 10% glycerol containing 500 nM heparin as a nonspecific competitor). When testing PNPase, final protein concentrations of approximately 0 to 1,000 nM were used, and the incubation was performed in TMK buffer (50 mM Tris-HCl [pH 7.5], 50 mM KCl, 10 mM [CH_3_COO]_2_Mg, 10% glycerol, 500 nM heparin). Reaction mixtures were incubated at 37°C for 1 h and then resolved by native electrophoresis in 0.5× Tris-borate-EDTA running buffer at 4°C for 3 h at 120 V using 5% polyacrylamide native gels. The gels were dried onto 3MM Whatman paper, exposed to a phosphor screen, and visualized using a Typhoon FLA 700 (GE Health Life Science). The signals were analyzed, and the equilibrium dissociation constant (*K_d_*) was determined as described earlier (60). Each EMSA was performed at least two times.

### Coimmunopurification of PNPase and RhlB.

pRADgro plasmids carrying His_6_ or His_6_-tagged PNPase and RhlB were transformed into the D. radiodurans R1 wild-type strain or D. radiodurans strains carrying 3×FLAG chromosomally tagged RhlB, RpsA, or Rho proteins. These strains (in biological triplicates) were grown to an OD_600_ of 0.8 and treated with formaldehyde (1% [wt/vol]) at room temperature for 15 min with continuous shaking to facilitate the cross-linking. Glycine was then added to a final concentration of 0.125 M to quench the cross-linking reaction. Cross-linked cells (50 mL) were washed three times with ice-cold binding buffer (20 mM Na_3_PO_4_, 300 mM NaCl, 10 mM imidazole, 1 mM MgCl_2_, and 20 mM KCl [pH 7.4] with 1 mM PMSF). Cells were then resuspended and lysed by sonication as described above. After lysis, the crude extracts were centrifuged at 12,000 rpm for 10 min at 4°C to collect the supernatant. For each His_6_-tag pulldown reaction, 1 mL of cell extracts containing 500 μg of protein was incubated with 50 μL of Ni-NTA magnetic beads (New England BioLabs) for 30 min at room temperature. The protein-protein mixtures were then washed using 500 μL of wash buffer (20 mM Na_3_PO_4_, 300 mM NaCl, and 20 mM imidazole [pH 7.4] with 1 mM PMSF) three times and eluted using 20 μL of elution buffer (20 mM Na_3_PO_4_, 300 mM NaCl, and 500 mM imidazole [pH 7.4] with 1 mM PMSF). To increase the yield, the elution step was repeated, and the eluates were combined. The protein samples were then concentrated by dialysis and subjected to LC-MS/MS or Western blotting. To determine whether the protein interactions are RNA-dependent, one set of samples was incubated with 2 μg of RNase A and 5 U of RNase T_1_ (Thermo Fisher Scientific) for 15 min at 22°C after cell lysis. coIP samples from cell lysates treated with or without RNase were subjected to Western blotting as described below.

### LC-MS/MS.

Proteins obtained from RNA affinity chromatography or coIP were resuspended in SDS sample buffer (0.5 M Tris-HCl [pH 6.8], 25% [vol/vol] glycerol, 0.5% [wt/vol] SDS, 0.5% [wt/vol] bromophenol blue, 0.05% [vol/vol] β-mercaptoethanol). The mixtures were denatured at 95°C for 5 min before being run ~3 mm into the 12% Mini-Protean TGX precast protein gels (Bio-Rad). After staining with Coomassie Bright Blue (Sigma-Aldrich), the protein bands were excised using a clean razor blade. The gel tubes were then dehydrated with 100% acetonitrile (Sigma-Aldrich), reduced with 10 mM dithiothreitol (DTT) for 30 min at room temperature, followed by alkylation with 50 mM iodoacetamide in the dark for another 30 min. The gels were washed, rehydrated, and then digested with 10 ng/μL trypsin (Thermo Fisher Scientific) overnight at 37°C. Proteins were then extracted from the gels using 5% formic acid and 1:2 (vol/vol) 5% formic acid-acetonitrile. The samples were sent for LC-MS/MS analysis at the University of Texas ICMB proteomics facility. The resulting protein spectral counts were searched against the UniProt D. radiodurans R1 (ATCC 13939) database using Sequest HT in Proteome Discoverer 1.4 and normalized to the total ion intensity. The identifications were validated with Scaffold v4.4.1 (Proteome Software) with >99.0% probability and with a minimum of two peptides at 99.0% peptide probability. The false discovery rate was set at 1% for peptides and 5% for protein identification. The difference between two strains or fractions was calculated by using a Student *t* test, and the *P* values were adjusted by the Benjamini-Hochberg method (61).

### Western Blotting.

Protein samples were resuspended in the SDS sample buffer, boiled at 95°C for 10 min, separated by 12% Mini-Protean TGX precast protein gels (Bio-Rad), and transferred to nitrocellulose membranes (Bio-Rad) using a Trans-Blot semidry transfer cell (Bio-Rad). Membranes were blocked with 5% milk powder overnight at 4°C and incubated for 1 h with monoclonal Anti-Flag M2-peroxidase antibody (Sigma-Aldrich, 1:1,000 dilution) or His_6_ tag monoclonal antibody (Thermo Fisher Scientific, 1:2,000 dilution) at room temperature. Afterward, the membranes were further incubated with anti-mouse IgG(H+L)/HRP conjugate (Promega) for another 1 h. After three washes with TBST, chemiluminescence was detected by using Clarity Western enhanced chemiluminescence substrate (Bio-Rad). Protein samples were visualized by Coomassie staining in parallel to show the equivalent protein loading in each lane. Quantitation of Western blot band intensities was done using the CLIQS software (TotalLab). Each Western blot analysis was performed at least three times.

### Measurement of cell survival.

D. radiodurans strains (WT, Δ*pnp*, Δ*rhlB*, and Δ*pnp *Δ*rhlB*) were grown to an OD_600_ of 0.8 and stressed with different dosages (0 to 200 mM) of H_2_O_2_ (Sigma-Aldrich) for 30 min at 4°C in the dark. The cells were serially diluted (10^−3^ to 10^−5^) with sterilized 1× phosphate-buffered saline and plated on TGY agar plates with appropriate antibiotics. The plates were incubated at 32°C for 3 days, and the colonies were counted. The relative survival was calculated as the percentage of the number of colonies under each H_2_O_2_ concentration compared to that for no H_2_O_2_ treatment.

### Determination of 8-oxoG-RNA and 8-oxoG-DNA levels.

D. radiodurans strains (WT, Δ*pnp*, Δ*rhlB*, and Δ*pnp *Δ*rhlB*) were grown to an OD_600_ of 0.8, stressed by 100 mM H_2_O_2_ (Sigma-Aldrich) for 30 min at 4°C in the dark, and then collected by centrifugation at 12,000 × *g* for 5 min. Total RNA was extracted and purified using the Direct-zol RNA miniprep kit (Zymo Research) according to the manufacturer’s protocol. DNA contamination in the RNA samples was removed by DNase digestion during the extraction. Total DNA was extracted using a Wizard genomic DNA purification kit (Promega), including an RNase digestion step to remove the RNA contamination in the samples. The levels of 8-oxoG in DNA and RNA samples were measured by a competitive ELISA (using a DNA/RNA oxidative damage EIA kit from Cayman Chemical) as described previously (62). Briefly, 1 μg of DNA or RNA from each sample was digested to nucleosides by nuclease P1 (Sigma-Aldrich) in 20 mM sodium acetate for 2 h at 37°C and dephosphorylated using the calf intestinal alkaline phosphatase (New England BioLabs) in a 100 mM Tris buffer (JT Baker) for 1 h at 37°C. Then, 1 μg of digested DNA or RNA samples was incubated with 8-oxoG antibody and tracers for 16 h. After extensive washes, the absorbance values were measured using a wavelength of 412 nm in the BioTek Cytation3 plate reader, and the results were analyzed as recommended by the manufacturer. All solutions used in this assay were nitrogen purged to ensure that no extra oxidation is introduced in the experiments.

### Determination of H_2_O_2_ content in *D. radiodurans* cell cultures.

D. radiodurans strains (WT, Δ*pnp*, Δ*rhlB*, and Δ*katA*) were grown to an OD_600_ of 0.8 and stressed by 0 or 100 mM H_2_O_2_ (Sigma-Aldrich) for 30 min at 4°C in the dark. The H_2_O_2_ level in the cultures was immediately determined using an Amplex Red kit (Thermo Fisher Scientific) as recommended by the manufacturer.

### Growth curve measurement.

Growth curves of D. radiodurans *strains* were evaluated using a Plate Reader (BioTek). Biological triplicates of each strain were distributed into 96-well plates with 200 μL TGY media. The initial OD_600_ was adjusted to 0.1, and the turbidity (600 nm) was measured every 30 min for 8 h as the cultures grew with shaking at 32°C. Catalase (Sigma-Aldrich) was added to a final concentration of 50 or 100 nM when needed.

### RNA helicase and degradation activity assay.

The combinational effect of PNPase and RhlB on structured RNA *in vitro* was performed as described previously (36). A 22-mer RNA oligonucleotide (5′-ACAGUAUUUGGUACUGCGCUCU-3′) was radiolabeled at the 5′ end using 10 μCi of [γ-^32^P]ATP (Perkin-Elmer) and T4 polynucleotide kinase (New England BioLabs). To generate dsRNA, the labeled 22-mer RNA oligonucleotide was incubated with an unlabeled 11-mer RNA oligonucleotide (5′-AGAGCGCAGUACC-3′, with complementary base pairs with the 22-mer oligonucleotide [underlined]) in RNA annealing buffer (10 mM Tris-HCl [pH 8.0], 20 mM KCl) by heating to 95°C for 5 min, followed by slow cooling (≫1 h) to room temperature. To maximize the hybridization efficiency, the 22-mer RNA oligonucleotide and the 11-mer RNA oligonucleotide were incubated at a ratio of 1:1.5 (mol/mol). 10 nM (final concentration) of double-stranded RNA duplex or 22-mer single-stranded RNA was incubated with PNPase (100 nM, final concentration) or RhlB (150 nM, final concentration) alone or with mixtures of PNPase and RhlB at 32°C for 30 min in 10 μL of activity buffer (20 mM Tris-HCl [pH 7.5], 5 mM MgCl_2_, 0.1 mM DTT, 5% glycerol, 10 mM K_2_HPO_4_, 50 mM KCl, and 3 mM ATP). The reaction was then stopped by adding 10 μL of 2× RNA loading buffer (New England BioLabs). The samples were separated on a 20% polyacrylamide native gel and visualized using Typhoon FLA 700 (GE Health Life Science).

### RT-qPCR.

RT-qPCR was performed using a Luna Universal One-Step RT-qPCR kit (New England Biolabs, Inc.) on a ViiA7 instrument (Applied Biosciences). cDNA was synthesized using 50 ng of RNA at 55°C for 10 min, followed by denaturation at 95°C for 1 min and 40 cycles of 95°C for 10 s and 60°C for 30 s. 16S rRNA was used as the internal normalization control. The relative expression of each gene was calculated using the 2^–ΔΔ^*^CT^* method (63). The experiments were performed in biological triplicate. The primers used for RT-qPCR are listed in Table S4.

### Statistics analysis.

Student two-sided t-tests were used to assess the significance of the results. Values of *P ≤  *0.05, 0.01, or 0.001 were considered to be statistically significant (*), highly significant (**), or extremely significant (***), respectively, as indicated by asterisks in the figures.

### Data availability.

The LC-MS/MS raw data have been deposited in the ProteomeXchange Consortium via the PRIDE partner repository (64) under the data set identifiers PXD028861 (RNA affinity chromatography) and PXD028866 (PNPase- and RhlB-coIP).
