# Supplementary material for: PNPase and RhlB Interact and Reduce the Cellular Availability of Oxidized RNA in Deinococcus radiodurans
Source: Microbiol Spectr. 2022 Jul 20;10(4):e02140-22. doi: 10.1128/spectrum.02140-22 (PMC9430589; doi:10.1128/spectrum.02140-22)
Supplement: Supplemental file 1 — Supplemental material. Download spectrum.02140-22-s0005.pdf, PDF file, 3.2 MB [file spectrum.02140-22-s0005.pdf]

Figure S1. **8-oxoG recognizing proteins identified by incubating 8-oxoG modified RNA with protein extracts of *D. radiodurans*.** (**A**) Denaturing gel of the proteins pulled down in the RNA affinity chromatography using the RNA oligonucleotide containing one 8-oxoG (8-oxoG ×1) and the unmodified control RNA oligonucleotide (Ctrl). The asterisks represent the bands that were pulled down by the 8-oxoG modified RNA oligonucleotide. (**B**) Principal component analysis (PCA) of proteins pulled down in the RNA affinity chromatography using the RNA oligonucleotide containing one 8-oxoG (8-oxoG ×1, in red) and the unmodified control RNA oligonucleotide (Ctrl, in blue). PC1 and PC2 represent the percentage of explained variances. Biological replicates are shown in different shape and spatial proximity among replicates suggest similar patterns of expression. (**C**) STRING-DB analysis of proteins significantly enriched in the RNA affinity chromatography using the RNA oligonucleotide containing one 8-oxoG compared to the unmodified control RNA oligonucleotide. RBPs that were chosen for further analysis are highlighted by red asterisks. DR_0550/MutT is highlighted by a purple asterisk. The line thickness indicates the strength of the interaction.

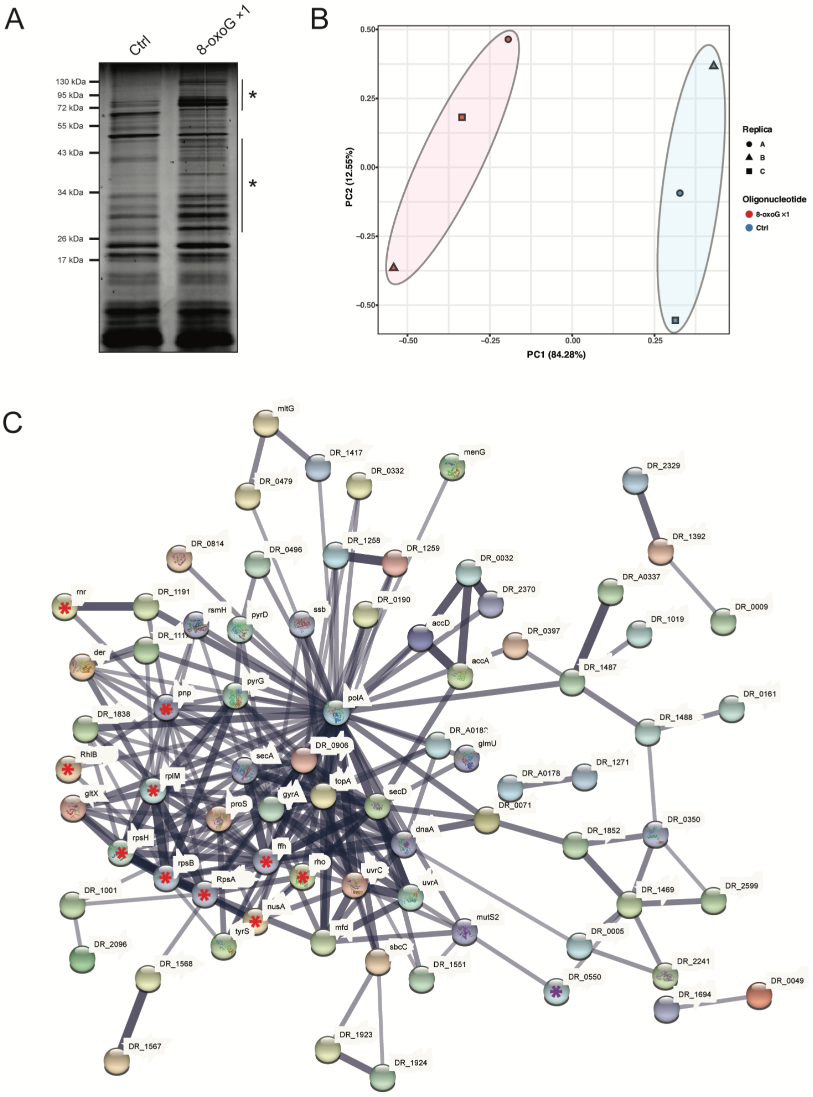

Figure S2. The binding of Rnr, NusA, and Ffh to the oligoribonucleotide carrying one 8-oxoG (8-oxoG ×1) and the control oligoribonucleotide (Ctrl). 500 ng of each purified recombinant protein with a His$_6$ tag was incubated with 10 µg of biotinylated oligoribonucleotide carrying one 8-oxoG (8-oxoG ×1) or the control oligoribonucleotide and then subjected to 500 µg Dynabeads™ MyOne™ Streptavidin C1 beads. After incubation, the beads were washed, and the proteins were resuspended in the SDS sample buffer. Western blotting was then performed to detect the proteins in the input and the pulldown fractions using an anti-His$_6$ antibody. 20% input, 20% of each recombinant protein applied to binding was also subjected to Western blotting.

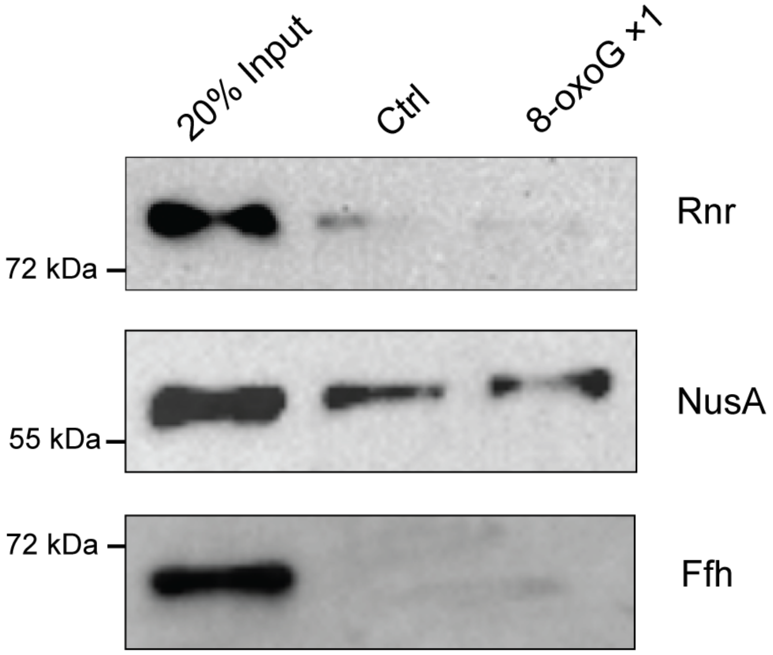

Figure S3. Representative EMSA figures to show the binding of PNPase, RhlB, Rho, and RpsA to 8-oxoG modified oligoribonucleotides (8-oxoG ×1-4) and the control oligoribonucleotide (Ctrl).

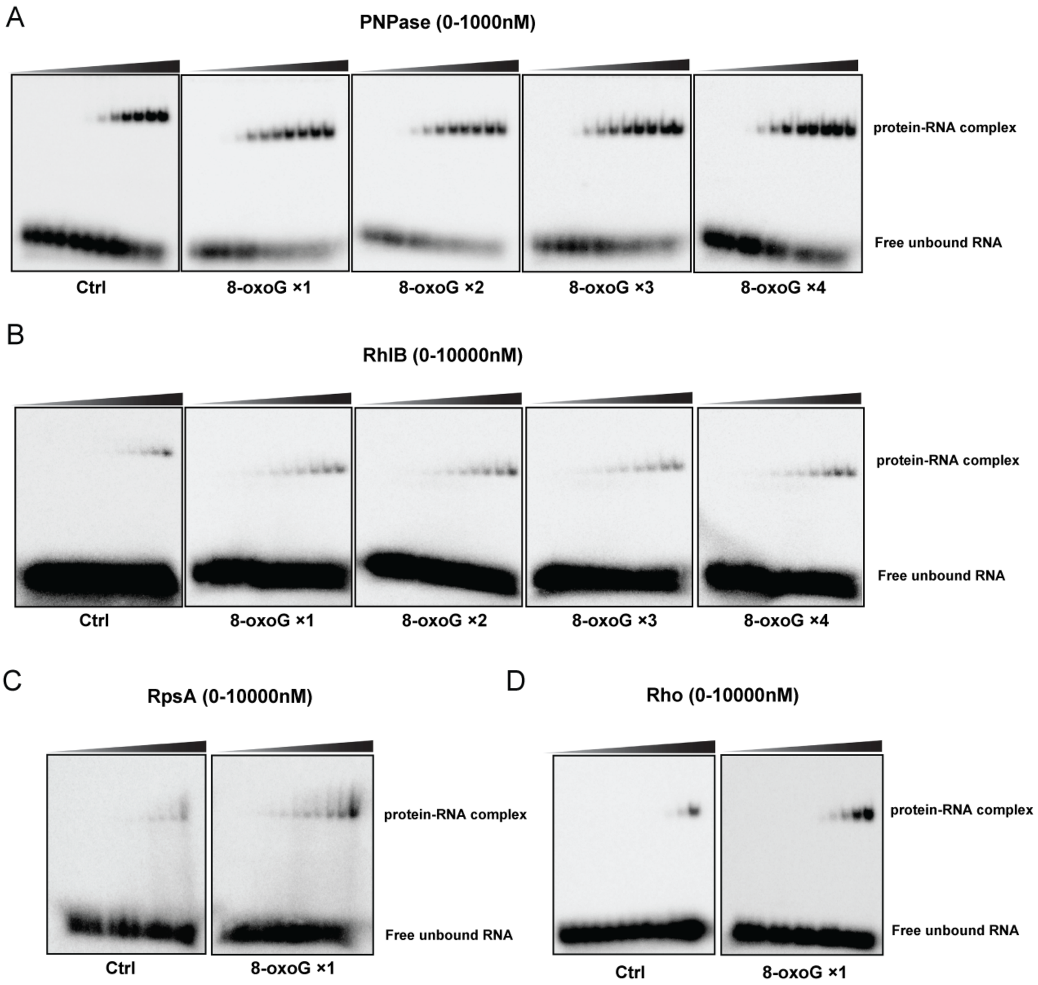

Figure S4. Confirmation and characterization of the gene knockout (KO) strains of *pnp* and *rhlB*. (**A**) PCR analysis of the wild type (WT) and KO strains confirmed the presence of the WT and mutant bands. (**B**) Quantification of relative *pnp* and *rhlB* levels in their respective KO strains as compared to the WT strain using RT-qPCR. (**C**) Growth of WT and KO strains in the presence or absence of 50 or 100 nM catalase in TGY medium at 32 °C. (**D**) Determination of H_2O_2 concentration in the cultures of WT and KO strains. The strains were grown to OD_{600nm} of 0.8 in TGY medium and treated with 100 mM H_2O_2 for 30 mins. The H_2O_2 concentration in the cell cultures was then measured using the Amplex Red kit. Data shown are the average of three biological replicates and Student's t-test was used to determine significance (***, $P \leq 0.001$). ns, not significant compared to the WT. (**E**) PCR analysis to examine DNA contamination in all the RNA samples (three replicates for each strain under each condition) used in the ELISA assay (Figure 7B). 50 ng of RNA from each sample was used as the template for PCR using specific primers for the gene encoding 16S rRNA. Two controls were also included (-DNase and +DNase) to prove the successful removal of DNA during the RNA extraction protocol used in this study (as shown in lanes 1 and 2). Lane 3 and 16, DNA ladders; Lanes 4-14, RNA samples extracted from the WT, Δ*pnp*, Δ*rhlB, and* Δ*pnp*Δ*rhlB* strains under 0 mM H_2O_2; Lanes 17-28, RNA samples extracted from the WT, Δ*pnp*, Δ*rhlB, and* Δ*pnp*Δ*rhlB* strains under 100 mM H_2O_2.

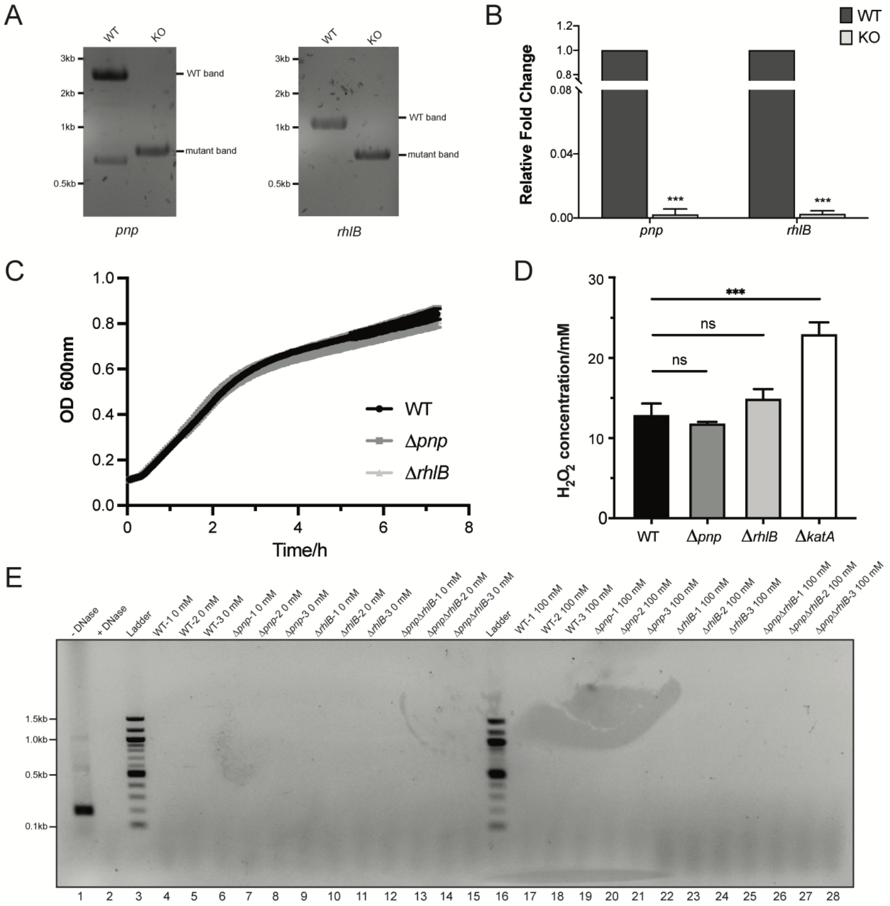

Figure S5. STRING-DB analysis of proteins significantly enriched in the PNPase-coIP. Proteins containing RNA binding domains are highlighted by red asterisks. The line thickness indicates the strength of the interaction.

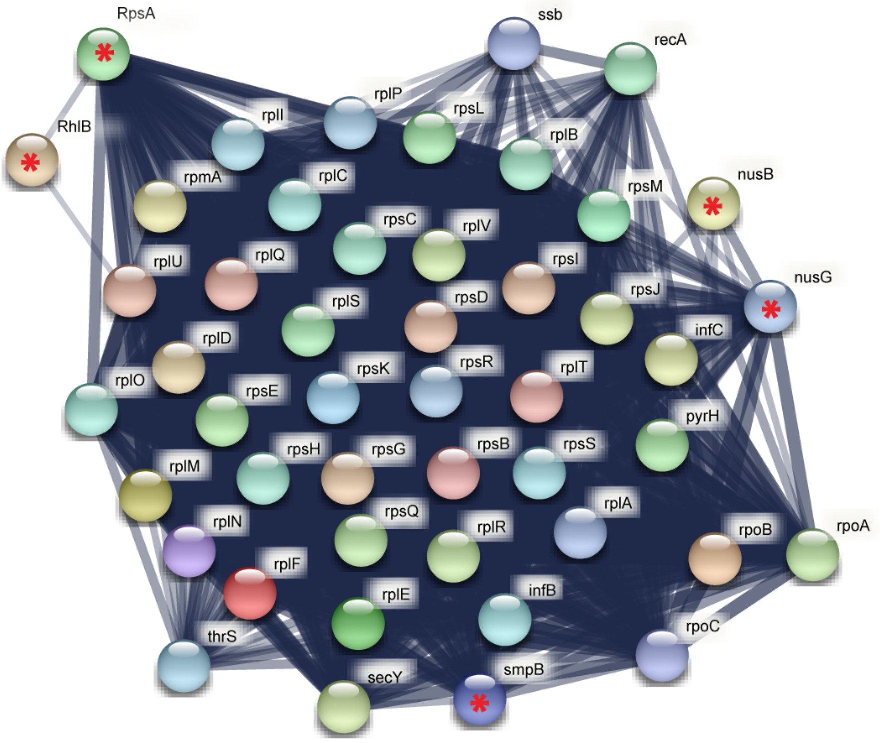

Table S1. Proteins significantly enriched in 8-oxoG-RNA affinity chromatography in *D. radiodurans*.

Table S2. Proteins significantly enriched in PNPase and RhlB co-immunoprecipitation in *D. radiodurans*.

Table S3. List of strains and plasmids used in this study.

Table S4. List of primers used in this study.
